# The Interplay between Glucose-Regulated Protein 78 (GRP78) and Steroids in the Reproductive System

**DOI:** 10.3390/ijms19071842

**Published:** 2018-06-22

**Authors:** Marylise Hebert-Schuster, Belinda Elisabeth Rotta, Brenna Kirkpatrick, Jean Guibourdenche, Marie Cohen

**Affiliations:** 1 INSERM U1139, Faculty of Pharmacy, Paris Descartes University, 75006 Paris, France; marylise.hebert-schuster@parisdescartes.fr (M.H.-S.); jean.guibourdenche@aphp.fr (J.G.); 2 Department of Automated Biological Diagnostic and Hormonology, Cochin Hospital, University Hospital Group of Paris-Centre (GHUPC, AP-HP), 75014 Paris, France; 3 Department of Gynecology Obstetrics, Faculty of Medicine, University of Geneva, 1205 Geneva, Switzerland; belinda.rotta@etu.unige.ch (B.E.R.); kirkpatrickbrenna@gmail.com (B.K.)

**Keywords:** GRP78, steroidogenesis, steroids, estrogen, testosterone, androgens, endoplasmic reticulum, ER stress

## Abstract

The glucose-regulated protein 78 (GRP78) is a molecular chaperone that is responsible for protein folding, which belongs to the heat shock protein 70 kDa (HSPA/HSP70). Because of the conjunction of GRP78 transcription with endoplasmic reticulum stress, the chaperone plays an important role in the unfolded protein response (UPR), which is induced after the accumulation of misfolded proteins. In the last years, a significant body of research concentrated on interplay between GRP78 and sexual steroid hormones. Throughout this review, we describe the mechanisms by which GRP78 regulates steroidogenesis at multiple levels and how steroids modulate GRP78 expression in different mammalian reproductive organs. Finally, we discuss the cooperation between GRP78 and steroids for cell survival and proliferation in the context of reproduction and tumorigenesis. This new paradigm offers significant opportunities for future exploration.

## 1. Introduction

Glucose-regulated protein 78 (GRP78), which is otherwise known as heat shock protein 70 kDa protein 5 (HSPA5) or immunoglobulin heavy chain-binding protein (BiP), is a molecular chaperone that facilitates proper protein folding, maintains proteins in a folded state, and prevents protein intermediates from aggregating in the endoplasmic reticulum (ER) [1,2]. GRP78 is a member of the HSPA/HSP70 family of proteins, and, like several other members of the HSPA family, performs a diverse array of functions in multiple cellular compartments, including the cytosol, nucleus, mitochondria, and cell surface [3,4]. Unlike other members of the HSPA family, however, GRP78 has a signal peptide sequence that causes it to reside primarily in the ER, and the synthesis of GRP78 is not significantly increased in heat shock conditions [2]. Rather, transcription of GRP78 is induced in conjunction with ER stress, which can be triggered by an array of biochemical imbalances, including calcium depletion in the ER lumen, hypoxic conditions, glucose deprivation, expression of mutant proteins, or the overexpression of wild-type proteins [4,5].

GRP78 plays a particularly important role in the unfolded protein response (UPR), which is induced when proteins are misfolded and subsequently accumulate in the ER [5]. The main pathways of the UPR are activated when GRP78 dissociates from the ER transmembrane proteins inositol-requiring enzyme 1 (IRE1), activating transcription factor 6 (ATF6), and protein kinase R-like endoplasmic reticulum kinase (PERK) [6,7,8]. The pathways of the UPR coordinate to both increase ER folding capacity by upregulating the ER folding mechanism ER-associated degradation (ERAD) and to decrease the ER folding load through selective mRNA degradation and translational repression [9]. When ER stress is particularly prolonged or intense, however, the UPR can activate downstream apoptotic pathways, such as the C/EBO homologous protein (CHOP) pathway, and lead cell to apoptosis [10]. Overexpression of GRP78 mitigates ER stress and resultant apoptotic effects by repressing the activity of the primary UPR transducers [6]. To enhance the elimination of misfolded proteins, GRP78 acts on the autophagic process at several points, from the initiation of autophagosomes formation to the cargo delivery of proteins for their degradation [11]. Moreover, GRP78 plays crucial functions in ER calcium storage that sustains Ca^2+^ homeostasis, and by this way contributes to modulation of several cellular processes, among which autophagy and mitochondrial function [12]. By forming complexes with apoptosis actors within the ER membrane, GRP78 promotes cell survival [13]. At the cell membrane, GRP78 functions as a signal-transducing receptor, initiating pro-survival signals, proliferation, cell migration, and invasion process [11]. Overall, in response to various cellular stresses, GRP78 ensure cytoprotective functions.

Steroid hormones can be split into two classes: corticosteroids (from the adrenal cortex) and sex steroids (from the gonads or the placenta). Within the sex steroids class are three types, according to the receptors to which they bind: androgens, estrogens, and progestogens. Estrogens and androgens play important roles in sexual differentiation and reproduction, particularly in the development and expression of male and female sexual characteristics. In addition to their reproductive function, sex steroid hormones perform important roles in regulating a wide range of physiological processes [14]. In this manner, sex steroids spatially and temporally coordinate extensive molecular networks that are involved in cellular proliferation, differentiation, motility, survival, and apoptosis. These effects are principally, but not only, mediated by specific receptors, the estrogen and androgen receptors (ESRs and ARs), which belong to the nuclear receptor superfamily.

Finally, the cellular functions of GRP78 and sex steroids appear to overlap and a significant body of research documents a link between GRP78 and sex steroid hormones. In the late 1980s, Li et al. suggested that GRP78 was a precursor for a theoretical steroidogenesis-activator polypeptide (SAP) and that GRP78 was constitutively expressed in steroidogenic cells [15]. Throughout this review, we seek to explore the reciprocal modulations of GRP78 and sex steroid hormones. We will examine the role of GRP78 in the production and action of sex steroids in the reproductive organs, including the testes, ovaries, and uterus, and conversely consider the effects of these hormones on GRP78 expression.

## 2. The Role of GRP78 in Steroidogenesis

**Figure 1:**
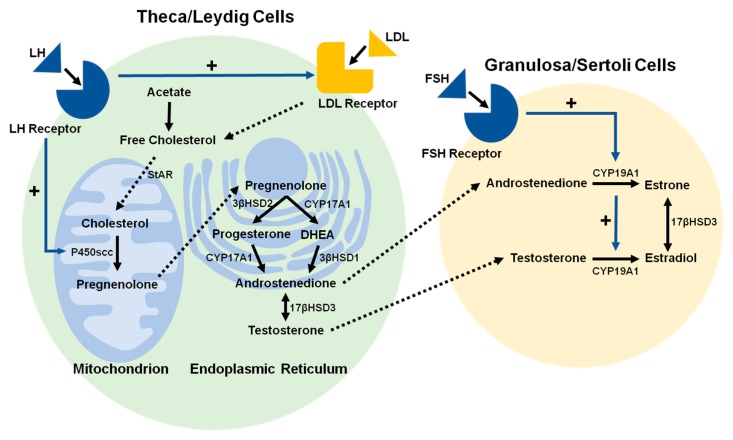
Schematic illustration of the process by which cholesterol is converted to steroid hormones in theca and granulosa cells in females and in Leydig and Sertoli cells in males. Free cholesterol is deposited into theca and Leydig cells by low-density lipoprotein (LDL) receptor-mediated LDL endocytosis. The conversion of cholesterol to pregnenolone is initiated by the binding of luteinizing hormone (LH) to the luteinizing hormone receptor (LHR), and conversion of androgens to estradiol (E_2_) is initiated by the binding of follicle-stimulating hormone (FSH) to the follicle-stimulating hormone receptor (FSHR). While the steroidogenic pathways in female and male reproductive organs are nearly identical, the principal hormone products of steroidogenesis differ in females and males, as indicated in the text. (Black solid line arrows: synthesis step; blue solid line arrow with + symbol: activation; dotted line arrows: transfer step).

The bulk of this chapter focuses on gonadal steroidogenesis. Although steroidogenesis occurs in different tissues, each individual steroidogenic reaction is generally catalyzed by the same steroidogenic enzyme, regardless of the tissue [16]. While the steroidogenic pathways in the ovaries and testes are essentially identical (Figure 1), the patterns of gonadal steroidogenesis are dictated by cell-specific expression of particular steroidogenic enzymes; abundant 17β-Hydroxysteroid dehydrogenase (17βHSD) in Leydig cells causes testosterone (T) to be the major product of testicular steroidogenesis, abundant aromatase (CYP19A1) in granulosa cells causes estradiol to be the major product of ovarian steroidogenesis during the follicular phase of the menstrual cycle, and abundant 3β-Hydroxysteroid dehydrogenase type 2 (3βHSD2) in the corpus luteum causes progesterone to be the major product of ovarian steroidogenesis during the luteal phase of the menstrual cycle [17].

As mentioned in the introduction, the first involvement of GRP78 in steroidogenesis was identified in rat Leydig cells in the late 1980s by Li et al. [15]. Through the isolation and sequencing of the genes encoding GRP78 from various species, including humans and rats, remarkable characteristics of GRP78 and other ER-localized proteins were observed: the acidic nature of the carboxyl end and the presence of the amino acid sequence lysine-aspartic acid-glutamic acid-leucine (KDEL) at the carboxyl termini, which is the signal for retention of non-membrane bound proteins in the ER [15,18]. After making this observation, Li et al. noticed that the carboxyl terminal sequence of rat GRP78 was nearly identical to that of SAP [15]. The primary role of SAP was postulated to be its promotion of the association of cholesterol with the side-chain cleavage cytochrome P450scc, an enzyme facilitating the conversion of cholesterol into pregnenolone [19]. Based on the findings of Pedersen et al., SAP likely played a role in facilitating the transport of cholesterol from the outer to the inner mitochondrial membrane. It was later hypothesized that the similarity between the carboxyl terminal sequence of GRP78 and SAP suggested their precursor-product relationship, which is supported by SAP’s similarities to mammalian GRP78, such as their shared isoelectric pH of 5.2 and affinity for ATP [15]. Li et al. (1989) concluded that SAP might arise from a proteolytic cleavage event after the hormone-regulated recruitment of a portion of GRP78 into the SAP precursor route. However, the fate of GRP78 during steroidogenesis remained unknown [15].

The most direct role, as currently described, of the chaperone protein GRP78 in steroidogenesis is its interaction with the steroidogenic acute regulatory protein StAR, which is an essential enzyme for steroidogenesis initiation [20]. StAR is responsible for the rate-limiting step in the production of steroid hormones, the transport of cholesterol from the outer to the inner mitochondrial membrane (IMM), where it is converted to pregnenolone by P450scc. To reach its final destination, StAR must change its conformation through multiple intermediate folding steps [20]. Prasad et al. showed that GRP78, through a transient interaction, facilitates StAR folding and the adoption of an active conformational state at the mitochondrial membrane [20]. In the absence of GRP78, StAR is not properly folded and thus proteolyzed. This result suggests that GRP78 plays an integral role in the function of StAR and in the initiation of steroidogenesis [20].

Similarly, some studies raised the importance of the chaperone role of GRP78 in the ovary. During the estrous cycle, ovulation is followed by a major transformation of the granulosa cells to form the corpus luteum and synthesize progesterone that is needed for an eventual pregnancy. This phenomenon requires a high level of cellular protein synthesis, which implies proper ER molecular chaperones function. Mizrachi and Segaloff had shown the ability of chaperone proteins in the ER to bind gonadotropin hormone receptors, follicle-stimulating hormone receptor (FSHR), and luteinizing hormone receptor (LHR), suggesting that the recruitment of GRP78 may be fundamental to the correct folding and transportation of gonadotropin receptors during biosynthesis [21]. The LHR and FSHR belong to the superfamily of G-protein-coupled receptors and play a role all along female reproductive phases. Proper LHR function is important because LHR mediates the actions of luteinizing hormone (LH) or human chorionic gonadotropin (hCG), without which steroidogenesis, ovulation and corpus luteum formation and persistence cannot take place [22,23]. After the LH surge and the subsequent ovulation, LHR expression at the cell surface is down-regulated, but a recovery is necessary for proper corpus luteum function. In rat granulosa cells, Kogure et al. highlighted an association of GRP78 with LHR [24]. In their experiments, GRP78 expression was induced by hCG and increased the LHR protein expression without affecting the LHR mRNA level, while it decreased FSHR, consequently inducing progesterone production [24]. Thereby, GRP78 participates in the recovery of LHR expression at the cell surface from down-regulation of the post-ovulation stage, which is necessary to corpus luteum formation and function. Finally, these studies suggest a functional link between GRP78 and granulosa cells life span during estrous cycle. As the LHR and FSHR share a high level of structural homology, the results raise the question of what determines the differential effects of the chaperone protein GRP78 on its targets.

**Figure 2:**
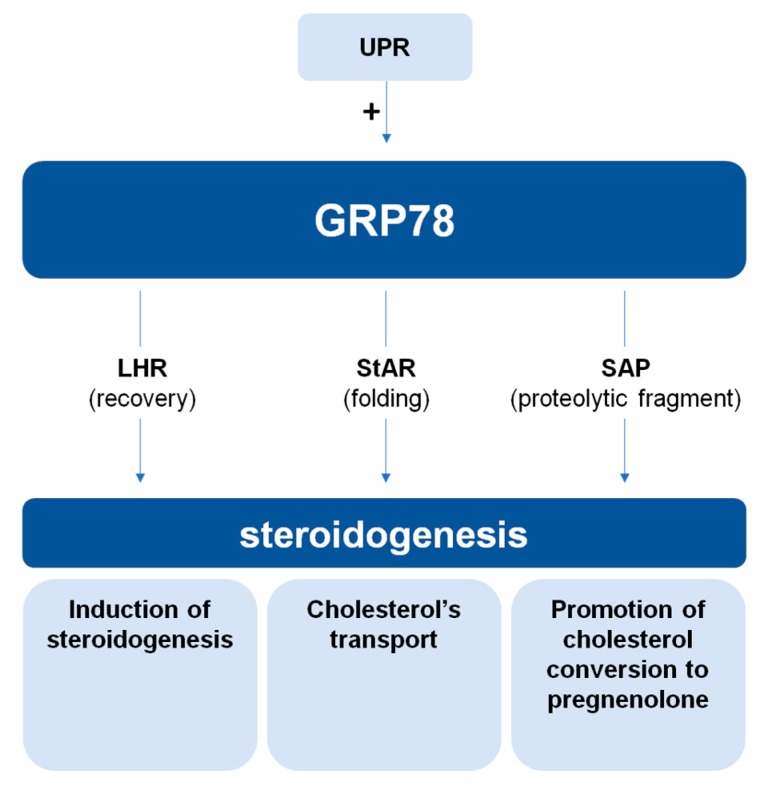
Schematic model of currently established interactions of GRP78 with steroidogenesis through three different pathways. In a preliminary scenario GRP78 promote the recovery of LHR and therefore initiates steroidogenesis and progesterone production. GRP78’s interaction with StAR ensures proper protein folding and subsequent activation of cholesterol transport. Furthermore, via the production of steroidogenesis-activator polypeptide (SAP), the conversion of cholesterol to pregnenolone is promoted. Meanwhile, an impact of the unfolded protein response (UPR) through the indirect role of GRP78 on steroidogenesis is implied. (“+”: activation).

In a general view, GRP78 appeared in multiple studies examining the relationship between the unfolded protein response (UPR) and steroidogenesis [25,26,27,28,29]. The UPR has been implicated as a regulator of steroidogenesis and, because GRP78 is heavily involved in this response, it is often used as a marker of the UPR. Using human and mouse ovarian and testicular tissues, it was observed that UPR has a negative effect on steroidogenesis, affecting StAR and decreasing progesterone and 3βHSD levels [25,26,27,28,29]. Interestingly, these observations suggest that the effects induced by the UPR response are contrary to those of GRP78, and are probably independent of GRP78. Indeed, two studies show that in leydig cells as in granulosa cells, ER stress-induced ATF6 pathway decreases steroid hormone synthesis, while increasing cell cycle arrest and apoptosis [25,28]. This is an interesting illustration of the subtle balance that is played out during the induction of an ER stress, of which GRP78 is a pivot, especially in secretory cells. GRP78 by its role of chaperone contributes to maintain a cellular homeostasis and to preserve the cell from death. But, if the imbalance exceeds the capabilities of GRP78, cell fate is reversed through UPR pathways, and physiological processes, such as steroidogenesis, are disabled. To conclude this second chapter, it appears that the chaperone protein GRP78 could regulate steroidogenesis in cells of different reproductive mammalian organs (Figure 2). Depending on the context, varying GRP78 expression levels can influence the expression of steroidogenic constituents, thereby implying the protein’s modulator role in different steroidogenesis-related pathways. Focusing on steroidogenesis as a general process, GRP78 plays a fundamental role in the induction of steroid hormone production. In fact, only through GRP78’s yield of SAP and its interaction with StAR, can steroidogenesis successfully begin. Furthermore, GRP78 seems to play an integral role in other steroidogenic steps through its interaction with LHR during the estrous cycle. Finally, ER stress, through GRP78 expression or induction of the UPR, adapts steroidogenic hormone production to cell condition. More investigations of the direct involvement and the molecular interactions of GRP78 with its targets should be performed.

## 3. The Role of Sex Steroids on GRP78 Expression and Function

In this third chapter, we focus on the inverse relationship of GRP78 with steroidogenesis, specifically on the effect of sexual steroids on GRP78 expression. First, we show the influence of estrogen on GRP78, analyzing reproductive process as well as tumorigenesis in reproductive organs. Then, we examine the impact of T on GRP78 expression in the context of spermatogenesis.

### 3.1. Estrogen

#### 3.1.1. Female Reproduction: Uterus and Early Pregnancy

Like other heat shock proteins, GRP78 plays different roles in all organs and along all steps of the reproductive process. Many studies have highlighted GRP78’s contribution to reproduction, implantation, and early embryogenesis. GRP78 in human endometrial and oviduct cells can bind to and may be involved in the conferring of fertilizing competence to spermatozoa [30]. After fertilization, the protein plays a role in facilitating cross-talk between the embryo and the uterus. At the embryo side, GRP78 is involved in inactivation and stabilization of p53 and in the regulation of trophoblastic invasion [31]. GRP78 may also regulate protein folding during placental development. On the maternal side, GRP78 may play a role in preparing a receptive endometrium by inducing endometrial cell apoptosis via UPR signaling and by generating space for the invading embryo [32]. A high level of GRP78 expression was detected in the decidualized area and in the glandular epithelium of mouse uteri during early pregnancy [32], suggesting that GRP78 could also contribute to uterine growth and to the transformation of uterine stromal cells into secretory decidual cells. Genomic studies in hamsters and mice showed that GRP78 is tightly regulated at the blastocyst implantation site [33].

Estrogen’s effects on the endometrium are of particular interest, as the endometrium is a dynamic tissue that undergoes hormone-dependent changes in cell differentiation and proliferation rates throughout the menstrual cycle. A dramatic upregulation of GRP78 by E_2_ has been observed in the uteri of ovariectomized mice in vivo and in uterine stromal murine cells in vitro, via an estrogen receptor (ESR) independent mechanism [34,35,36]. Upregulation of HSPs, including GRP78 by estrogen and estrogenic chemicals, such as Bisphenol A (BPA), has also been described in mouse uteri and fibroblast cells in an estrogen receptor-dependent way [37,38,39]. The modulation of GRP78 upon E_2_ stimulation must be related to the complex functions of endometrial cells and the spatiotemporal physiological changes that are required during the estrous cycle and embryo implantation. Lin et al. reported that GRP78 expression and distribution in the murine uterus and oviducts vary with the phases of the estrous cycle [36]. GRP78 predominately localizes to the uterine luminal and glandular epithelial cells during the estrous phase, when the estrogen levels peak. Conversely, Guzel et al. reported that GRP78 immunoreactivity was significantly higher in endometrial glandular and stromal cells during phases of the menstrual cycle when E_2_ levels were known to be the lowest [1]. These discrepancies could be attributed to interindividual variations of protein expression level. We were not able, in a similar study of human endometrial biopsies, to determine the rate of variation of GRP78 expression during the estrous cycle [40]. A longitudinal study should be designed to avoid interindividual variations.

#### 3.1.2. Tumorigenesis: Endometrial, Ovary and Breast Cancer

Invasion of the decidua by trophoblastic cells resembles the invasion of cancer cells [31], and GRP78 mediates both cell growth and invasiveness in endometrial cancer [31,41]. More generally, UPR has been extensively studied in reproductive tissue cancers, and GRP78 expression, as part of this process, has been linked to the survival and proliferation of cancer cells [42]. To ensure the proper folding of the increased protein levels that is required for cell proliferation, cells must increase chaperone levels.

Otherwise, estrogens, via the estrogen receptor α (ESRα), are known to stimulate cell proliferation and tumor growth. Estrogens’ growth-stimulating properties are illustrated by endocrine therapies (mainly for breast and ovary tumors) that are based on inhibitors that target estrogens and their receptors to avoid tumor cells proliferation [43]. Recent studies have highlighted that, in ESR+ cancer cells (estrogen receptors expressed at the cell surface), a weak and sustained estrogenic activation of the UPR permits cell proliferation and resistance to therapy [44]. Through this mode of UPR activation, called the “anticipatory” mode by some authors, cancer cells preactivate the UPR in absence of ER stress, in anticipation of future increased protein folding required for proliferation [44]. E_2_ bind to ESRα, which activates a phosphorylated phospholipase C γ (PLCγ)-UPR pathway, involving ATF6. Chronic UPR activation leads to elevated intracellular calcium level, which is a proliferation signal, and increases the expression of ER chaperones, such as GRP78 [44,45]. This process primes tumor cells to growth phase and inherent accumulation of unfolded protein, and protects cells from subsequent UPR-mediated apoptosis [45]. But, the GRP78 overexpression may also contribute to anti-estrogen resistance [46]. In human breast cancer cells, high levels of GRP78 confer resistance to estrogen starvation-induced apoptosis via an inhibition of the BCL2-interacting killer (BIK), a pro-apoptotic BH3-only protein, and the induction of three anti-apoptotic molecules: B-cell lymphomas BCL2, BCL-XL, and BCL-W. Moreover, the overexpression of GRP78 markedly increases microtubule-associated proteins 1A/1B light chain 3B (LC3II) protein levels and decreases nucleoporin p62 protein levels, indicating increased autophagy [47]. These results highlight E_2_-induced GRP78 expression as part of resistance mechanisms to therapies, and GRP78 as a potential therapeutic target.

### 3.2. Testosterone

#### 3.2.1. Male Reproduction: Testis

It is both surprising and regrettable that few studies exist on GRP78 induction by steroids in the testis. However, GRP78 is expressed in almost all cell types of the seminourous tubules, except in spermatogonia and in mature ejaculated human spermatozoa [48,49]. Strong immunostaining of GRP78 was observed in the cytoplasm surrounding the nuclei of spermatocytes and round spermatids. In epididymis, GRP78 displays different subcellular localizations depending on the tissue zone, suggesting different roles in this secretory tissue [49]. Lachance team’s results indicate that GRP78 and other HSPs may play important roles in the process of spermatogenesis. We mentioned above the binding of endometrial GRP78 to spermatozoa during capacitation, a process that may represent a possible conditioning cross-talk between male gametes and the maternal host organism [30]. Hence, the potential T-dependent regulation of GRP78 in the male genital tract and gametes is of interest, but there is a shortage of addressing this topic. In the late 80s, in conjunction with the first publications characterizing GRP78, Day et al. investigated the mechanism by which the expression of the *HSPA5* gene (GRP78 protein coding) is regulated using a mouse Sertoli cell line [50]. Treatment of Sertoli cells with T enhanced GRP78 transcription levels in a dose-dependent manner [50]. The endocrine disruptor BPA also induces GRP78 mRNA expression in Sertoli cells [51]. Finally, GRP78 seems to play an important role in spermatogenesis and fertilization, but its putative regulation by T in the male genital tract and gametes requires further investigations.

#### 3.2.2. Tumorigenesis: Prostate Cancer

Another element of the genital tract for which a link between T and GRP78 has been established is the prostate. Several recent studies have focused on ER stress in prostate cancer cells [52]. Alongside surgery, the standard therapeutic option that is used to treat prostate cancer is androgen ablation, which leads to an initial regression of the tumor in most cases. As a major reproductive secretory organ, the prostate is particularly reliant on proper functioning of the ER and is vulnerable to agents or conditions that cause ER stress. *Androgen receptor* (*AR*) gene and UPR-related genes expressions, including *HSPA5* expression, are correlated in prostate cancer cell [53,54], suggesting interplay between the UPR and androgens. Indeed, GRP78 is increased by androgen treatment in LNCaP cells [53]. In our experiments on both malignant (LNCaP, DU145 and PC3) and non-malignant (PNT1B and PNT2C2) prostate cell lines, we also observed an upregulation of GRP78 mRNA and protein levels after T treatment in non-malignant cells that express AR (PNT1B) and in malignant cells independently of their AR status [55]. We then treated DU145 (AR+ malignant cells) and PNTB1 (AR+ non-malignant cells) with T-BSA (Testosterone linked to Bovine Serum Albumin). GRP78 expression was only increased in DU145 after treatment, confirming that T-induced GRP78 expression is, at least in part, independent of AR status in malignant cells [55]. Finally, these preliminary results suggest that, as described above for E_2_, T could promote cells survival and proliferation of cancer cells in part via UPR and GRP78 expression. This process could allow for the tumor to temporary adapt to cell stress, probably via the modulation of autophagy and cell death [56]. Understanding the precise mechanisms requires further study and may lead to the identification of new therapeutic targets.

In conclusion of this third chapter, molecular biological insights from the past decade have uncovered a ubiquitous regulation of GRP78 by steroids in reproductive organs. Depending on the tissue and the pathophysiological context of GRP78 induction, GRP78 levels can either be increased or decreased by the action of steroids. In order to carry out its functions, GRP78 is modulated not only temporally but also spatially, especially during the menstrual cycle. Moreover, in vitro and in vivo mouse uterus models highlighted that GRP78 regulates the functions of ESRα by interacting with the receptor on a molecular level [34]. GRP78 was shown to be one of the four most abundant HSP70s to associate with ESRα and to regulate the assembly, trafficking, and transcriptional activity of the receptor and to regulate E_2_-mediated cell proliferation [57]. Hence, it seems that after the estrogen-induced expression in endometrial cells, GRP78 is required for the maintenance of the gene transcription function of ESRα and may be critical for coordinating the estrogen-elicited endometrial response [34]. By this process, GRP78 seems to act as a critical booster of estrogenic potency and uterine growth. Interestingly, the study of the weak xenoestrogen kepone illustrated the substantial facilitation provided by GRP78 to estrogen functions under sub-optimal estrogenic induction, as the administration of even low doses of kepone leads to uterine growth thanks to GRP78/ESRα cooperation [35]. With regard to these data, the interplay of GRP78 with the estrogenic pathway seems to be from steroidogenesis to E_2_ action. Finally, the understanding of the precise mechanisms of steroids/GRP78 partnership could open perspectives for the prevention, treatment, and follow up of various diseases of hormone-sensitive organs.

## 4. Conclusions

GRP78 acts as a multifaceted protein at the cross-talk of many cellular processes. As an ER chaperone, it facilitates proper folding of steroidogenesis intermediaries. As part of the ER stress and UPR signaling, it participates in the regulation of protein production and cell fate. It is therefore not surprising that, in hormone-sensitive tissues, GRP78 is targeted by steroids to regulate cell proliferation, survival, or death. Studies to date have demonstrated that, in reproductive tissues, E_2_ and T are able to induce the expression of GRP78 in healthy and tumor cells, although the precise mechanisms remain unclear. In cancer tissues, the “anticipatory” UPR activation by mild and transient E_2_ stimulation is of particular interest as it increases GRP78 expression and protects cells from stress-related death. The implication of this pathway in resistance to therapies opens up promising prospects. Overall, whether in healthy cells or tumors, these processes seem to be great assets for the main cellular action of steroids: the promotion of growth and survival. By the regulation of the UPR, and consequently, the overexpression of GRP78, steroids can inhibit the apoptotic signaling and promote cell survival and growth. In return, the ability of GRP78 to regulate StAR, the acute regulator of steroidogenesis, and LHR allows for a positive feedback loop on steroids synthesis and action. Finally, we demonstrate a complex but functional interplay of ER chaperone GRP78 and steroid hormones, working together for cell survival and proliferation in the context of reproduction and tumorigenesis. As ER stress is a common constitutive feature of different types of cancers [58], this field of research offers significant opportunities for future exploration.

## Abbreviations

AR	Androgen Receptor
ATF6	Activating Transcription Factor 6
BPA	Bisphenol A
CHOP	C/EBO Homologous Protein
E_2_	Estradiol
ER	Endoplasmic Reticulum
ESR	Estrogens Receptor
FSH	Follicule-Stimulating Hormone
FSHR	Follicule-Stimulating Hormone Receptor
GRP78	Glucose-Regulated Protein 78
hCG	Human Chorionic Gonadotropin
HSP	Heat Shock Protein
IRE1	Inositol-Requiring Enzyme 1
LDL	Low-Density Lipoprotein
LH	Luteinizing Hormone
LHR	Luteinizing Hormone receptor
PERK	Protein Kinase R-like Endoplasmic Reticulum Kinase
SAP	Steroidogenesis-Activator Polypeptide
StAR	Steroidogenic Acute Regulatory Protein
T	Testosterone
UPR	Unfolded Protein Response
3βHSD2	3β-Hydroxysteroid dehydrogenase type 2
17βHSD	17β-Hydroxysteroid dehydrogenase

## References

[1] Guzel E., Basar M., Ocak N., Arici A., Kayisli U.A. (2011). Bidirectional interaction between unfolded-protein-response key protein HSPA5 and estrogen signaling in human endometrium. Biol. Reprod..

[2] Lee A.S. (2005). The ER chaperone and signaling regulator GRP78/BiP as a monitor of endoplasmic reticulum stress. Methods.

[3] Stetler R.A., Gan Y., Zhang W., Liou A.K., Gao Y., Cao G., Chen J. (2010). Heat shock proteins: Cellular and molecular mechanisms in the central nervous system. Prog. Neurobiol..

[4] Ni M., Zhang Y., Lee A.S. (2011). Beyond the endoplasmic reticulum: Atypical GRP78 in cell viability, signalling and therapeutic targeting. Biochem. J..

[5] Kaufman R.J. (1999). Stress signaling from the lumen of the endoplasmic reticulum: Coordination of gene transcriptional and translational controls. Genes Dev..

[6] Bertolotti A., Zhang Y., Hendershot L.M., Harding H.P., Ron D. (2000). Dynamic interaction of BiP and ER stress transducers in the unfolded-protein response. Nat. Cell Biol..

[7] Schroder M., Kaufman R.J. (2005). ER stress and the unfolded protein response. Mutat. Res..

[8] Shen J., Chen X., Hendershot L., Prywes R. (2002). ER stress regulation of ATF6 localization by dissociation of BiP/GRP78 binding and unmasking of Golgi localization signals. Dev. Cell.

[9] Gardner B.M., Pincus D., Gotthardt K., Gallagher C.M., Walter P. (2013). Endoplasmic reticulum stress sensing in the unfolded protein response. Cold Spring Harb. Perspect. Biol..

[10] Zhang L.H., Zhang X. (2010). Roles of GRP78 in physiology and cancer. J. Cell. Biochem..

[11] Casas C. (2017). GRP78 at the Centre of the Stage in Cancer and Neuroprotection. Front. Neurosci..

[12] Kania E., Pajak B., Orzechowski A. (2015). Calcium homeostasis and ER stress in control of autophagy in cancer cells. BioMed Res. Int..

[13] Lee A.S. (2014). Glucose-regulated proteins in cancer: Molecular mechanisms and therapeutic potential. Nat. Rev. Cancer.

[14] Ogino Y., Tohyama S., Kohno S., Toyota K., Yamada G., Yatsu R., Kobayashi T., Tatarazako N., Sato T., Matsubara H. (2018). Functional distinctions associated with the diversity of sex steroid hormone receptors ESR and AR. J. Steroid Biochem. Mol. Biol..

[15] Li X.A., Warren D.W., Gregoire J., Pedersen R.C., Lee A.S. (1989). The rat 78,000 dalton glucose-regulated protein (GRP78) as a precursor for the rat steroidogenesis-activator polypeptide (SAP): The SAP coding sequence is homologous with the terminal end of GRP78. Mol. Endocrinol..

[16] Miller W.L. (1988). Molecular biology of steroid hormone synthesis. Endocr. Rev..

[17] Miller W.L., Auchus R.J. (2011). The molecular biology, biochemistry, and physiology of human steroidogenesis and its disorders. Endocr. Rev..

[18] Munro S., Pelham H.R. (1987). A C-terminal signal prevents secretion of luminal ER proteins. Cell.

[19] Pedersen R.C., Brownie A.C. (1983). Cholesterol side-chain cleavage in the rat adrenal cortex: Isolation of a cycloheximide-sensitive activator peptide. Proc. Natl. Acad. Sci. USA.

[20] Prasad M., Pawlak K.J., Burak W.E., Perry E.E., Marshall B., Whittal R.M., Bose H.S. (2017). Mitochondrial metabolic regulation by GRP78. Sci. Adv..

[21] Mizrachi D., Segaloff D.L. (2004). Intracellularly located misfolded glycoprotein hormone receptors associate with different chaperone proteins than their cognate wild-type receptors. Mol. Endocrinol..

[22] Menon K.M., Gunaga K.P. (1974). Role of cyclic AMP in reproductive processes. Fertil. Steril..

[23] Hsueh A.J., Adashi E.Y., Jones P.B., Welsh T.H. (1984). Hormonal regulation of the differentiation of cultured ovarian granulosa cells. Endocr. Rev..

[24] Kogure K., Nakamura K., Ikeda S., Kitahara Y., Nishimura T., Iwamune M., Minegishi T. (2013). Glucose-regulated protein, 78-kilodalton is a modulator of luteinizing hormone receptor expression in luteinizing granulosa cells in rats. Biol. Reprod..

[25] Park S.J., Kim T.S., Park C.K., Lee S.H., Kim J.M., Lee K.S., Lee I.K., Park J.W., Lawson M.A., Lee D.S. (2013). hCG-induced endoplasmic reticulum stress triggers apoptosis and reduces steroidogenic enzyme expression through activating transcription factor 6 in Leydig cells of the testis. J. Mol. Endocrinol..

[26] Park H.J., Park S.J., Koo D.B., Lee S.R., Kong I.K., Ryoo J.W., Park Y.I., Chang K.T., Lee D.S. (2014). Progesterone production is affected by unfolded protein response (UPR) signaling during the luteal phase in mice. Life Sci..

[27] Kim J.H., Park S.J., Kim T.S., Kim J.M., Lee D.S. (2016). Testosterone production by a Leydig tumor cell line is suppressed by hyperthermia-induced endoplasmic reticulum stress in mice. Life Sci..

[28] Xiong Y., Chen H., Lin P., Wang A., Wang L., Jin Y. (2017). ATF6 knockdown decreases apoptosis, arrests the S phase of the cell cycle, and increases steroid hormone production in mouse granulosa cells. Am. J. Physiol. Cell Physiol..

[29] Takahashi N., Harada M., Hirota Y., Zhao L., Azhary J.M., Yoshino O., Izumi G., Hirata T., Koga K., Wada-Hiraike O. (2017). A Potential Role for Endoplasmic Reticulum Stress in Progesterone Deficiency in Obese Women. Endocrinology.

[30] Lachance C., Bailey J.L., Leclerc P. (2007). Expression of Hsp60 and Grp78 in the human endometrium and oviduct, and their effect on sperm functions. Hum. Reprod..

[31] Arnaudeau S., Arboit P., Bischof P., Shin-ya K., Tomida A., Tsuruo T., Irion O., Cohen M. (2009). Glucose-regulated protein 78: A new partner of p53 in trophoblast. Proteomics.

[32] Lin P., Jin Y., Lan X., Yang Y., Chen F., Wang N., Li X., Sun Y., Wang A. (2014). GRP78 expression and regulation in the mouse uterus during embryo implantation. J. Mol. Histol..

[33] Lei W., Herington J., Galindo C.L., Ding T., Brown N., Reese J., Paria B.C. (2014). Cross-species transcriptomic approach reveals genes in hamster implantation sites. Reproduction.

[34] Ray S., Hou X., Zhou H.E., Wang H., Das S.K. (2006). BiP is a molecular link between the phase I and phase II estrogenic responses in uterus. Mol. Endocrinol..

[35] Ray S., Xu F., Li P., Sanchez N.S., Wang H., Das S.K. (2007). Increased level of cellular BiP critically determines estrogenic potency for a xenoestrogen kepone in the mouse uterus. Endocrinology.

[36] Lin P., Chen F., Yang Y., Song Y., Li X., Lan X., Jin Y., Wang A. (2012). GRP78 expression and immunohistochemical localization in the female reproductive tract of mice. Theriogenology.

[37] Papaconstantinou A.D., Fisher B.R., Umbreit T.H., Goering P.L., Lappas N.T., Brown K.M. (2001). Effects of beta-estradiol and bisphenol A on heat shock protein levels and localization in the mouse uterus are antagonized by the antiestrogen ICI 182,780. Toxicol. Sci..

[38] Papaconstantinou A.D., Goering P.L., Umbreit T.H., Brown K.M. (2003). Regulation of uterine hsp90α, hsp72 and HSF-1 transcription in B6C3F1 mice by β-estradiol and bisphenol A: Involvement of the estrogen receptor and protein kinase C. Toxicol. Lett..

[39] Kita K., Jin Y.H., Sun Z., Chen S.P., Sumiya Y., Hongo T., Suzuki N. (2009). Increase in the levels of chaperone proteins by exposure to β-estradiol, bisphenol A and 4-methoxyphenol in human cells transfected with estrogen receptor α cDNA. Toxicol. In Vitro.

[40] Cohen M. (2014). Human endometrial biopsies study. Unpublished data.

[41] Cali G., Insabato L., Conza D., Bifulco G., Parrillo L., Mirra P., Fiory F., Miele C., Raciti G.A., di Jeso B. (2014). GRP78 mediates cell growth and invasiveness in endometrial cancer. J. Cell. Physiol..

[42] Guzel E., Arlier S., Guzeloglu-Kayisli O., Tabak M.S., Ekiz T., Semerci N., Larsen K., Schatz F., Lockwood C.J., Kayisli U.A. (2017). Endoplasmic Reticulum Stress and Homeostasis in Reproductive Physiology and Pathology. Int. J. Mol. Sci..

[43] Bertelli G., Paridaens R. (2006). Optimal sequence of hormonotherapy in advanced breast cancer. Curr. Opin. Oncol..

[44] Shapiro D.J., Livezey M., Yu L., Zheng X., Andruska N. (2016). Anticipatory UPR Activation: A Protective Pathway and Target in Cancer. Trends Endocrinol. Metab..

[45] Andruska N., Zheng X., Yang X., Helferich W.G., Shapiro D.J. (2015). Anticipatory estrogen activation of the unfolded protein response is linked to cell proliferation and poor survival in estrogen receptor α-positive breast cancer. Oncogene.

[46] Fu Y., Li J., Lee A.S. (2007). GRP78/BiP inhibits endoplasmic reticulum BIK and protects human breast cancer cells against estrogen starvation-induced apoptosis. Cancer Res..

[47] Cook K.L., Shajahan A.N., Warri A., Jin L., Hilakivi-Clarke L.A., Clarke R. (2012). Glucose-regulated protein 78 controls cross-talk between apoptosis and autophagy to determine antiestrogen responsiveness. Cancer Res..

[48] Huo R., Zhu Y.F., Ma X., Lin M., Zhou Z.M., Sha J.H. (2004). Differential expression of glucose-regulated protein 78 during spermatogenesis. Cell Tissue Res..

[49] Lachance C., Fortier M., Thimon V., Sullivan R., Bailey J.L., Leclerc P. (2010). Localization of Hsp60 and Grp78 in the human testis, epididymis and mature spermatozoa. Int. J. Androl..

[50] Day A.R., Lee A.S. (1989). Transcriptional regulation of the gene encoding the 78-kD glucose-regulated protein GRP78 in mouse sertoli cells: Binding of specific factor(s) to the GRP78 promoter. DNA.

[51] Tabuchi Y., Takasaki I., Kondo T. (2006). Identification of genetic networks involved in the cell injury accompanying endoplasmic reticulum stress induced by bisphenol A in testicular Sertoli cells. Biochem. Biophys. Res. Commun..

[52] Storm M., Sheng X., Arnoldussen Y.J., Saatcioglu F. (2016). Prostate cancer and the unfolded protein response. Oncotarget.

[53] Sheng X., Arnoldussen Y.J., Storm M., Tesikova M., Nenseth H.Z., Zhao S., Fazli L., Rennie P., Risberg B., Waehre H. (2015). Divergent androgen regulation of unfolded protein response pathways drives prostate cancer. EMBO Mol. Med..

[54] Tan S.S., Ahmad I., Bennett H.L., Singh L., Nixon C., Seywright M., Barnetson R.J., Edwards J., Leung H.Y. (2011). GRP78 up-regulation is associated with androgen receptor status, Hsp70-Hsp90 client proteins and castrate-resistant prostate cancer. J. Pathol..

[55] Cohen M. (2011). Prostate cancer cell lines study (PNT1B, PNT2C2, LNCaP, DU145, PC3). Unpublished data.

[56] Bennett H.L., Fleming J.T., O’Prey J., Ryan K.M., Leung H.Y. (2010). Androgens modulate autophagy and cell death via regulation of the endoplasmic reticulum chaperone glucose-regulated protein 78/BiP in prostate cancer cells. Cell Death Dis..

[57] Dhamad A.E., Zhou Z., Zhou J., Du Y. (2016). Systematic Proteomic Identification of the Heat Shock Proteins (Hsp) that Interact with Estrogen Receptor α (ERα) and Biochemical Characterization of the ERα–Hsp70 Interaction. PLoS ONE.

[58] Urra H., Dufey E., Avril T., Chevet E., Hetz C. (2016). Endoplasmic Reticulum Stress and the Hallmarks of Cancer. Trends Cancer.

